# The Effect of Granular Activated Carbon and Biochar on the Availability of Cu and Zn to *Hordeum sativum* Distichum in Contaminated Soil

**DOI:** 10.3390/plants10050841

**Published:** 2021-04-22

**Authors:** Marina Burachevskaya, Saglara Mandzhieva, Tatiana Bauer, Tatiana Minkina, Vishnu Rajput, Victor Chaplygin, Aleksey Fedorenko, Natalia Chernikova, Inna Zamulina, Sergey Kolesnikov, Svetlana Sushkova, Leonid Perelomov

**Affiliations:** 1 Academy of Biology and Biotechnology, Southern Federal University, Stachki Ave. 194/1, 344090 Rostov-on-Don, Russia; marina.0911@mail.ru (M.B.); msaglara@mail.ru (S.M.); bauertatyana@mail.ru (T.B.); otshelnic87.ru@mail.ru (V.C.); afedorenko@mail.ru (A.F.); nat.tchernikova2013@yandex.ru (N.C.); inir82@mail.ru (I.Z.); kolesnikov1970@list.ru (S.K.); terra_rossa@mail.ru (S.S.); 2 Faculty of Natural Sciences, Tula State Lev Tolstoy Pedagogical University, Lenin Ave. 125, 300041 Tula, Russia; perelomov@rambler.ru

**Keywords:** soil, heavy metals, granular activated carbon, biochar, immobilization, spring barley, remediation

## Abstract

The presence of heavy metals in the soil could impose serious problems on soil-plant systems due to the accumulation of heavy metals in plants. Even vital elements such as Cu and Zn have a toxic effect in the case of excessive intake by living organisms. The present work aimed to investigate the content of loosely bound (exchangeable, complexed, and specifically sorbed) compounds of Cu and Zn and their availability to spring barley (*Hordeum sativum* distichum) in contaminated Haplic Chernozem soil under the conditions of a model experiment (five approximate permissible concentrations (APC) and 10 APC of metal). Changes in the bioavailability of the metals upon application of carbon sorbents were observed. An increase in loosely bound metal compounds has been shown under conditions of soil contamination with metals (up to 57% of the total content). The increase in the availability of Cu in the soil was mainly due to the formation of complexed metal forms with organic matter (up to 17%). The availability of Zn was found to be associated with an increase in exchangeable (up to 21%) and specifically sorbed compounds (up to 27%). Granular activated carbon (GAC) and biochar have high sorption properties. A decrease in the content of loosely bound compounds of metals was established, especially in the most mobile forms such as exchangeable and complexed forms. The introduction of sorbents into the soil opened up a new venue for binding heavy metals in situ, eventually leading to a decrease in their bioavailability. The inactivation of Cu and Zn in the soil upon the application of sorbents led to a decrease in metal absorption by spring barley. The highest efficiency of biochar application was established at a dose of 2.5% and 5% in soil contaminations of 5 APC and 10 APC of Cu or Zn. The efficiency of the use of sorbents was more influenced by the dose of application than by the type of sorbent. There was no significant difference between biochar and GAC. Stabilization and inactivation of metals may improve soil fertility and plant growth.

## 1. Introduction

Soil contamination with various types of chemical compounds has been a growing concern in recent years. Among these pollutants, heavy metals (HMs) are of remarkable environmental importance and biological significance [1,2]. Due to the fact that HMs are non-degradable by chemical and microbial decomposition, HMs are persistent and hard to remove once released into soils. Trace elements including Cu and Zn at high concentrations in the environment may exert toxic effects. Zinc and copper are priority soil pollutants in the Rostov region of south Russia [3].

HM immobilization is the most common in situ remediation method for contaminated soils, and it is achieved by adding sorbents and/or ameliorating additives to allow HM stabilization, which leads to a decrease in their bioavailability in the soil. This has found extensive application in advanced remediation technologies for the restoration and cleaning up of polluted sites [4]. In addition, the choice of sorbents should be based not only on their capacity to bind HM compounds strenuously, but also on creating an ideal environment for plant growth and development. In this case, both the barrier functions of soils and plants can be increased to attain the optimal effect of their application. A wide range of materials are used as sorbents, i.e., natural mineral and organic substances, industrial and agricultural waste, as well as especially developed materials [5,6].

Carbon sorbents have been considered desirable for the immobilization of HMs due to their eco-friendly nature. Among various carbon sorbents, activated carbon and biochar are the most commonly used sorbents for soil remediation [6]. Heat treatment (pyrolysis) of plant materials and various industrial wastes can be used to attain carbon sorbents (granular activated carbon (GAC) and biochar). Pyrolysis is the thermal decomposition of organic-matter-containing waste in the absence of an oxidizing agent, which results in the production of charcoal-like residue containing high boiling resin-like compounds, and is performed using pyrolysis gas [7]. Carbon sorbents can firmly bind the soil pollutants, which is facilitated by their tremendously high stability in the soil [1,8,9], and their great sorption capacity [6,10,11]. Hence, the half-period of biochar mineralization (T50) is reported to reach 100 years or more, and the mineralization rate of GAC is even lower [12].

The specific pore surface area of carbon sorbents is large and has a range between 400–2000 m^2^/g, with a sorption capacity of 200–980 cmol/kg [13]. Due to their large specific surface area, rich porous structure, and broad range of surface functional groups, carbonaceous adsorbents have been widely used in HM sorption [14]. GAC is one of the most effective means of decontaminating polluted soil. However, it differs from other carbon sorbents in the stage of activation following pyrolysis.

Activation permits the attainment of a sorbent with a pore area of about 1000–1500 m^2^ per gram. These extreme values account for the exceptionally high efficiency of activated carbon and are also comparable with several organic and inorganic pollutants. Activated carbon (AC) is an ideal material for removing contaminants [15].

Biochar also deserves attention along with GAC due to its specific structural characteristics, economic feasibility, and environmental sustainability [16]. However, the high cost and complexity of the production of GAC should be noted, whereas biochar can be produced from agricultural waste, sewage, and other waste. The immobilization of contaminants using biochar is becoming increasingly common.

Biochar is a black carbon obtained by thermal degradation processes, through which residual biomass is converted into a charred material at temperatures between 300 °C and 700 °C [17]. Biochar consists of a variety of materials that cause differences in their properties, as well as the resulting sorbent quality [18,19,20,21]. However, the quality of the resulting biochar depends on the feedstock. Substantial attention by researchers has been paid to altering the properties of biochar depending on the pyrolysis temperature regime [22]. Wei et al. and Cimo et al. concluded that increased pyrolysis temperature could result in biochar with a higher carbon content [23,24].

Biochar has properties of chemical inertness and stability [25]. The natural degradation of biochar itself is very slow, with an expected half-life of 102–107 years for biochar C in the environment [26]. Due to these features, it is widely known as an effective sorbent for contaminated soil remediation. Recent studies have shown that biochar offers an excellent ability to remove inorganic pollutants in water and soil systems. It has been widely recognized that biochars exhibit great affinity for HMs due to their abundance of functional groups (e.g., phenolic, hydroxyl, and carboxyl groups) and their porous structure [27,28]. It was shown that the application of rice straw and bamboo wood biochar in sandy loam paddy soil significantly reduced the concentration of mobile forms of HMs, i.e., Cd, Cu, Pb, and Zn [29]. Zhan et al. applied biochar to farmland around a Pb-Zn mining area (Yunnan Province, China), and found a significant reduction in the contents of available Pb and Zn in the soil and their contents in the plants [30]. Research has also shown that biochar has positive effects on the metal removal capacity and growth response of hyperaccumulators, particularly promoting the growth of those species that are capable of accumulating Cd, Cu, and Pb [31,32]. Although many studies have been conducted in recent years in both laboratory and field conditions, the influence of carbon sorbents on the availability of HMs in the soil requires further research [33,34]. The results of carbon sorbent introduction into the soil are not always unambiguously positive due to their different properties [35,36].

Overall, findings on the effects of carbon sorbents have been made either in acidic soils or poor sandy soils. Nevertheless, the impact of the properties and effectiveness of carbon sorbents are of great interest, especially their utilization in Chernozems, which are the most fertile soils in the world. At the same time, this type of soil is subjected to intense anthropogenic pressure. Haplic Chernozem is widely spread in the steppe zone of Southern Russia and is favorable for crop production, which highlights the importance of its investigation [37]. In the south of Russia, the share of the industrial output is high, including the metallurgical, chemical, coal and ore mining industries, as well as the largest enterprises of the energy and oil refining industries. The proximity of industry and agriculture brings the problem of soil fertility and quality and, as a result, the cultivation of environmentally friendly products to the fore.

The present investigation aimed to establish the impact of carbon sorbents (GAC and biochar) on the availability of Cu and Zn and their availability to plants in Haplic Chernozem soil.

## 2. Objects and Methods

### 2.1. Study Objects

In order to study the effect of carbon sorbents, a model experiment with artificial soil contamination was carried out. The study included samples from the humus-accumulative A1 horizon of Haplic Chernozem (Rostov region, Rostov-on-Don, Russia). The studied soil was characterized by the following properties: pH—7.3; 48.1% silt (particles with a diameter of 0.002–0.02 mm), 28.6% clay (particles with a diameter of <0.002 mm), the content of organic carbon was 3.7%; carbonates—0.1%; CEC soil—36 cmol (+)/kg. Haplic Chernozem soil has good physical and chemical properties: water and air permeability, loose composition, and high moisture capacity, high content of organic matter, and the presence of highly dispersed micellar forms of carbonates. Chernozem has mainly good physical and chemical properties [38]. At the same time, the introduction of carbon sorbents improves these properties on soil with a high clay content [39].

To construct the experiment, plastic vessels with a volume of 2.5 L were used, into which 2 kg of prepared soil was added. To do this, large organic residues were extracted from the samples with tweezers, and the soil was ground in a porcelain mortar with a pestle and sifted through a sieve with a cell diameter of 2 mm. Then, Cu and Zn acetate salts were separately added into the soil in each vessel and thoroughly mixed. Cu(CH_3_COO)_2_·H_2_O and Zn(CH_3_COO)_2_·2H_2_O were purchased from Joint Stock Company “Khimreaktiv” (Nizhny Novgorod, Russia) with a purity >99%, produced according to Russian State standards [40]. These salts were selected because of their good solubility and their capacity for quick and complete interaction with the soil mass. Acetate anions are the natural products of plant metabolism and cannot significantly change the nutrient regime of the soil. HM doses 5 and 10 times higher than approximate permissible concentrations (APC) of metals were selected. For Cu, APC is 132 mg/kg and for Zn APC is 220 mg/kg [41]. These concentrations imitated the levels of soil pollution in the impact zones of the Rostov region by these metals, and other regions near chemical plants and non-ferrous metal ore mining and processing enterprises [42,43]. Experiments were conducted with the temperature ranging from 25 °C ± 5 °C (day) to 18 °C ± 2 °C (night) in normal lighting mode in aerobic conditions. Each treatment was prepared in triplicate. The design of the experiments and the sampling procedure were carried out as per the standard methodology [44,45]. The homogeneity of the distribution of metal salts in the soil was ensured by thorough mixing. The risk of salt leaching into the lower part of the vessel was prevented by watering with a spray gun on top of the vessel and by using lower watering through the tube during the entire period of the experiment.

To reduce Cu and Zn bioavailability for plants, after two months of metal incubation, sorbents were added to the contaminated soil according to the scheme outlined in Table 1, ground up with a pestle, and thoroughly mixed with the soil. The homogeneity of the sorbents in the soil was assessed visually. The following sorbents were used: GAC from birch wood waste (a commercialized material acquired from Vekton corporation) (brand BAU-A) [46] and biochar from birch wood waste (a commercialized material acquired from Ivchar company) (brand A) with a carbon content of at least 85%, which allowed us to compare the efficiency of their use. Two months later, GAC and biochar were added to the contaminated soil in the amount of 1%, 2.5%, and 5% of the soil mass as sorbents. Previous research demonstrated that two months are sufficient time for the transformation process of pollutants in Haplic Chernozem soil [47].

**Figure 1:**
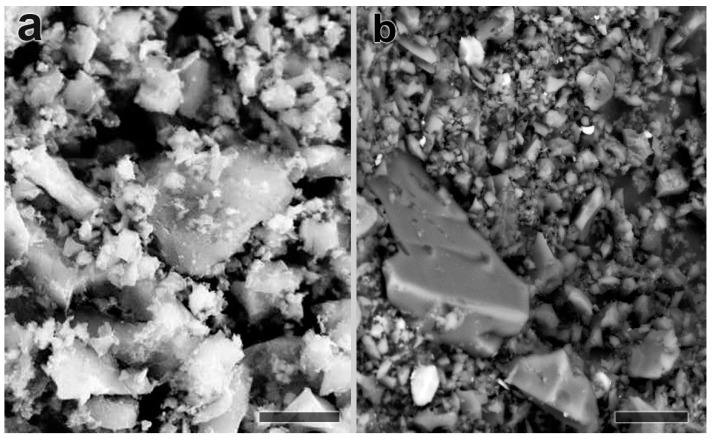
Surface microstructure of biochar (**a**) and granular activated carbon (**b**).

Characteristics of the surface and morphology of the used sorbents were analyzed by means of scanning electron microscopy (SEM) (Carl Zeiss EVO-40 XVP SEM microscope). The biochar sample was a multi-layer, highly porous material with a large surface area (Figure 1a). The size and structure of the particles repeated the morphology of the original plant material from which this sample was made. The particles of biochar had a predominantly flat elongated irregular shape. From the sagittal perspective, conglomerates of carbon flakes with sizes ranging from 10 to 100 µm were visible. A well-developed sample surface with many convexities and invaginations was observed.

The GAC sample consisted of particles with a size of 0.1–1 mm (Figure 1b) and was homogeneous. Most of the material was loose and grainy. The particles were predominantly isomorphic and fragmented. The total weight of the samples can be divided into skeletal and finely dispersed components. Large particles ranging in size from 10 to 100 microns were covered with small particles less than 10 microns in size. The finely dispersed component consisted of both aggregates and individual particles. The surface of the particles was smooth and invagination was not observed.

The used carbon sorbent’s porosity and specific surface area were determined using an ASAP 2020 volumetric analyzer, considering the low-temperature nitrogen adsorption method. The surface and porosity parameters of the carbon sorbent were calculated using the Brunauer–Emmett–Teller (BET) method according to N_2_ in the equilibrium value range P/P0 = 0.05–0.33. The BET method allowed the measurement of the specific surface area of the materials. The specific surface area was calculated via BET according to S_N2_. The volumes of micro- and mesopores (V_mic_ and V_mes_), the total limiting volume of adsorption space (V_S_) and the limiting volume of the adsorption space of micropores (W_O_), the characteristic adsorption energy in micropores (E), and the half-width of the slit-like model of micropores (X) were calculated. To assess the relative content of micro- and mesopores available for the adsorption of substances dissolved in water, the adsorption of marker substances such as iodine and methylene blue dye from aqueous solutions was carried out in addition to standard methods [48,49]. The total contents of C, H, and N in the carbonaceous sorbents were determined using a CHN elemental analyzer (TOC-L CPN Shimadzu, Japan). The ash content in the sorbents was measured by burning the adsorbents at 650 °C for 3 h, and the O content was calculated based on mass difference. Biochar was found to be a microporous material with a small surface area of mesopores (0.04 cm^3^/g), and a considerable surface area of micropores (0.60 cm^3^/g) (Table 2).

GAC, on the other hand, showed developed microporosity and a significant content of mesopores (0.17 cm^3^/g). The total pore volume of the sorbents differed slightly. The differences in surface area were due to differences in manufacturing technology: the GAC activation process allows for a larger surface area. However, the specific surface area of wood biochar was also quite high. The share of moisture in the sorbents was estimated according to the Russian State standard [50]. The content of moisture in GAC was 3.2%. In biochar made of wood, the share of moisture was 3.6%. For the GAC, the ash content was 18.0%. Biochar had an ash content of 15.2%. The sorbents had the pH values of 9.3 for GAU and 9.1 for biochar. The values of moisture, pH, and ash content of the two sorbents were slightly different, which could be explained by the similar properties of the original wood raw materials. Biochar had slightly higher H/C and O/C molar ratios than GAC. This indicates the greater aromaticity of biochar. The higher polarity index ((N + O)/C ratio) in biochar also indicates a larger number of polar oxygen-containing groups.

After metals were incubated with sorbents for 2 months, two-row spring barley (*Hordeum sativum* distichum) was sown. Spring barley is one of the main staple crops grown on the territory of the Rostov region. The spring barley is the fourth largest globally produced grain crop, and has been used as a bioindicator to assess the impact and accumulation of pollutants [47,51]. Liu et al. used barley as a bioindicator of Cd pollution in the range of 30–120 mg/L to detect the DNA damage induced by Cd [29]. It was also considered to assess the soil toxicity, especially mutagenicity [52].

There was no pre-treatment of seeds. The seed germination energy was measured, and found to be high (99%). Twelve caryopses were sown per vessel at 2 cm deep according to the scheme of the experiment (Table 1) and thinned to 10 plants after 2 weeks. The seeding rate was calculated according to Russian standards [47,53,54]. No additional fertilizers and pesticides were applied during the experiment.

The vegetation (period from 23 May to 15 August 2020) took place outdoors under natural light conditions. The daytime temperature ranged from 20 °C–35 °C and the nighttime temperature ranged from 12 °C–27 °C. The photoperiod ranged from 14 h 13 min to 15 h 56 min. The atmospheric humidity ranged from 19% to 94%. The vessels were covered by a shed to avoid rainfall. An optimal humidity of 60% of the total moisture capacity was maintained in the soil during the entire course of the experiment. The moisture was maintained by adding distilled water through vertical pipes from a bottle to the top in the center of the vessel to avoid the leaching of metal. The position of the vessels was changed randomly every week. After the growing season, plant samples were taken simultaneously with soil samples.

### 2.2. Analytical Methods

The plants were harvested at the maturity stage phase (day 85 after sowing). The plant parts (stems, roots, grains) were dried at 105 °C for 30 min and then at 70 °C to a constant weight to determine dry biomass. The roots were washed before drying and grinding. For the determination of Cu and Zn in plants, an air-dried 1 g sample was ashed in a muffle furnace at 450 °C for 6 h. The ash was dissolved in 5 mL of 20% HCl and filtered, then moved to a 50-mL flask. The mineralization of the plant samples was carried out according to the standard Russian method [55]. The accuracy of the metal content was verified with a state-standard sample of the composition of barley grain (GSO ZYa-01) (All-Russian Research Institute of Agrochemistry).

After the harvesting of plants, the soil samples were taken and dried to an air-dry condition. Soil samples weighing 5 g were ground in a porcelain mortar with a pestle and sifted through a sieve with a cell diameter of 1 mm. The total content of Cu and Zn in the soil was determined via the X-ray fluorescence method using a X-ray spectrometer (Spectroscan MAX-GV, SPECTRON Ltd., Saint-Petersburg, Russia). The accuracy of the metal content was verified with reference state standard soil sample no. 9288–2009 (Federal State Unitary Enterprise Ural Research Institute of Metrology) to control measurement errors relating to the total content of certified components in the soil. Duplicates and reagent blanks were also used as a part of quality control. The correctness of the obtained results was repeatedly confirmed by international intercalibrations.

To characterize the pool of metals that can migrate from the soil into the adjacent media, including plants and natural waters, the loosely bound compounds of HMs were determined [56]. This group of the loosely bound compounds of HMs includes exchangeable, complexed and specifically sorbed forms [43]. Three parallel extracts were used: (1) 1 M CH_3_COONH_4_, pH 4.8; (2) 1% EDTA in CH_3_COONH_4_; and (3) 1 M HCl. The ratio of solid phase:solution in the preparation of the extracts was 1:10. The first solution includes exchangeable HM compounds. The displacement of cations occurs at the expense of the participation of protons (pH 4.8) and ammonium ions; additionally, acetate ions bind the replaced cations in the soluble complexes [56]. The second solution extracts exchangeable HMs and those bound in the organometallic complexes. The difference between the metal contents in the first and second extracts characterizes the content of metals in complexes within the soil organic matter. In the third solution, specifically adsorbed metals were determined together with the exchangeable metals. A considerable part of the specifically adsorbed HM was relatively loosely fixed by iron, aluminum, and manganese oxides and hydroxides and by carbonates. The main effect of HCl is the acid hydrolysis of the soil sample. A portion of the hydrogen ions is spent in the neutralization of carbonates and a part is spent in the replacement of exchangeable cations [56]. The differences between their amounts represent specifically adsorbed HM compounds. The HM content in the extracts was determined by atomic absorption spectrophotometry (AAS, KVANT 2-AT, Kortec Ltd., Moscow, Russia). The wavelength of 218.7 nm was used for Zn and 213.8 nm was used for the analysis of Cu on AAS. Calibration curves for Cu and Zn at AAS were obtained simultaneously using aqueous standard solutions and the generalized standard addition method. Analytical data quality of metal was ensured by using Environmental Protection Agency (EPA) samples in water and the results were found to be within the prediction intervals. The standard reference material (SRM) of Cu and Zn (Certipur^®^ standards for AAS, Merck KGaA Company, Darmstadt, Germany) was used for the calibration and quality assurance for each analytical batch. The obtained values of 1 mg/L standard reference material were 0.98 mg/L for Cu and 1.01 mg/L for Zn. The uncertainty was estimated to be ±5%.

The chemicals used in the study were high purity chemical substances, and all the solutions were prepared with ultrapure water (resistivity of 18.25 MU/cm). All acids (HCl and CH_3_COOH) used in this study were purified via sub-boiling distillation. Ammonium acetate powder was dissolved in water to obtain a 1 M solution.

The group of loosely bound compounds of HMs is the most mobile and available for plants. There is a close correlation between the content of mobile forms of metals in the soil and their content in plants [3,57]. The parallel extraction scheme allows the identification of the most important compounds from an ecological point of view. This group includes exchangeable, complexed, and specifically sorbed metal compounds. The loosely bound compounds of HMs are absorbed by plants and can migrate to other adjacent environments [58]. To characterize the availability of HMs in the soils, we calculated the portion of mobile forms (exchangeable, complexed, and specifically sorbed forms) as a percentage of the total content. The portions of these mobile forms within the group of loosely bound compounds were also calculated.

To assess the accumulation by spring barley under polluted conditions, the accumulation coefficient (AC) was calculated. It indicates the degree of bioavailability of elements, and its change indicates the anthropogenic load on the soil. The AC is represented by the ratio of the element concentration in the dry mass of a plant (roots) to the content of its mobile compounds in the soil [59,60]. The loosely bound compounds of HMs in soils were used as mobile ones [57]; if AC < 1, the root system barrier function was observed.

### 2.3. Statistical Analysis

All the analyses were performed in triplicate using STATISTICA 10.0 software, and the averages with standard deviations are presented. Determination of the statistical significance of differences between the mean values was performed using the Mann–Whitney U-test, and the values were considered significant at a significance level of *p* < 0.05.

## 3. Results and Discussion

### 3.1. Cu and Zn Speciation and Bioavailability in Soil

The total content of Zn was 84.3 ± 6.1 mg/kg and of Cu was 45.3 ± 6.1 mg/kg in Haplic Chernozem soil (Table 3). These values correspond to the background values for these metals [61].

**Figure 2:**
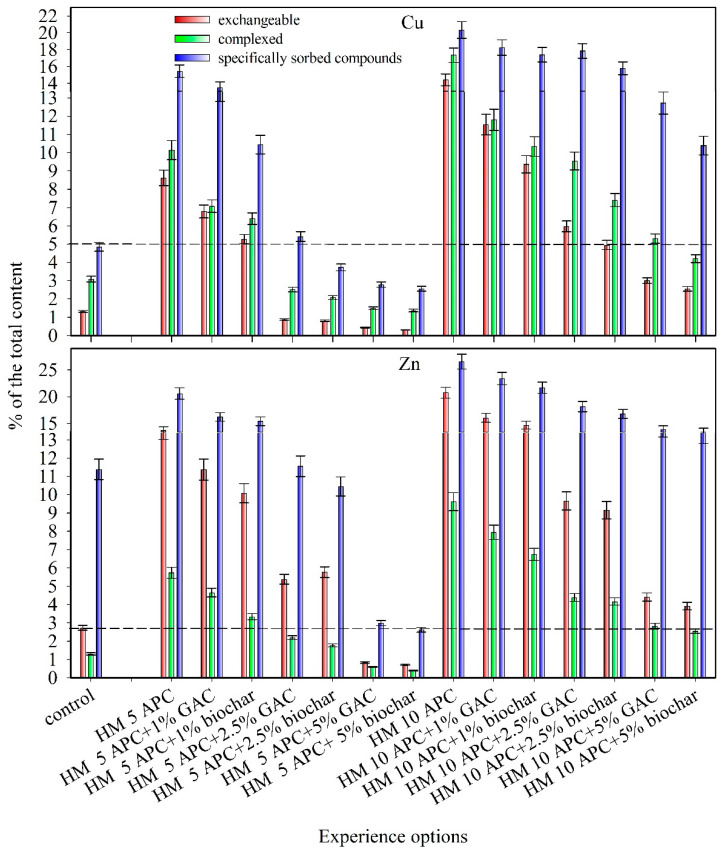
The proportion of loosely bound compounds of Cu and Zn in the soil of the model experiment under contamination with doses of 5 and 10 approximate permissible concentrations (APC) of heavy metal (HM), % of the total content.

The percentage of loosely bound compounds of Cu and Zn in the studied soil was determined after the plants were harvested. The content of the exchangeable form of HMs was lower than 5% of the total content (Table 3, Figure 2). Together with the total content of HMs, the content of exchangeable, complexed, and specifically sorbed forms also increased when Cu and Zn were added into the soil. Simultaneously, the differences between the average values of the content of loosely bound compounds between the variants with soil contamination with HMs and without contamination were statistically significant at the *p*-level of 0.05. Thus, the content of loosely bound compounds increased. Under Zn contamination of the soil, the largest increase in the concentration of exchangeable metal forms was observed (up to 21% at a pollution dose of 10 APC), reflecting the tendency of Zn to induce ion exchange in the soil.

Loosely bound compounds of metals were mainly represented by their specifically sorbed forms. This tendency was observed in both polluted and unpolluted soil (Table 3). With pollution, the percentage of the most mobile forms increased—exchangeable forms (by 8%–13% for Cu contamination and 11%–18% for Zn contamination) and complexed forms (by 7%–14% for Cu contamination and 5%–9% for Zn contamination) (Figure 2). These results can be attributed to the differences in metal characteristics and resultant affinity to sorption sites. Previous studies demonstrated that the analysis of the near edge structure XANES confirmed the organophilicity of Cu with organic ligands [63]. It was shown that interaction between Cu^2+^ ions and humic acids of soils might lead to the origin of octahedral inner-sphere chelate complexes. Soil-adsorbed Cu^2+^ cations can partly substitute octahedrally coordinated Al^3+^ ions in clay minerals. Copper can also be adsorbed in the form of a Cu–Cu dimer by silicate and/or aluminosilicate groups that are incompletely coordinated or possess structural defects (bond lengths and double bonds) [63].

Zinc has a weak complexing ability with organic matter [64,65]. One gram of humic acids was able to bind 0.995 mEq Zn and 2390 mEq Cu, whereas the content of the first element in the system was twice higher than that of the second one [48]. Studies have shown that Zn was more liable to interact with the mineral components of the soil [64,66,67].

The share of loosely bound compounds of HMs decreased with the introduction of carbon sorbents. When GAC was added to the samples, they demonstrated a decrease in the loosely bound metal compounds. Statistically significant differences between the average values of the content of loosely bound metal compounds were observed even with the introduction of carbon sorbents at a dose of 1% (at *p*-level <0.05). Copper exchangeable forms decreased by 2%–11%, complexed forms by 3%–12%, and specifically sorbed forms by 2%–12%. Zinc exchangeable forms were reduced by 3%–17%, Zn complexed forms by 1%–7%, and Zn specifically sorbed forms up to 4%–18%.

There was a decrease in the loosely bound compounds of both metals: 4%–11% for Cu exchangeable forms, 4%–13% for Cu complexed forms, 5%–12% Cu specifically sorbed forms, and 4%–17% for Zn exchangeable forms, and 3%–7% for Zn complexed forms, 6%–18% for Zn specifically sorbed forms when biochar was introduced into the soil (Figure 2).

Furthermore, the average values of the content of loosely bound Cu compounds in contaminated and uncontaminated soil either did not have statistically significant differences or were significantly lower in the variants of the experiment with the introduction of 2.5% sorbents into the contaminated soil at 5 APC. The same effect in reducing Zn compounds was observed with the introduction of carbon sorbents at a dose of 5%. The introduction of sorbents into the contaminated soil with Zn or Cu in the amount of 10 APC significantly reduced the proportion of loosely bound compounds of HMs in the soil. However, these parameters remained significantly higher than in the soil without the introduction of pollutants (Figure 2).

The metal availability decreased with an increase in the dose of carbon sorbents (GAC and biochar). This rule was observed for both metal contaminants (Table 3). In the variants contaminated with 5 APC of metal, the content of the HM exchangeable forms did not exceed the MPC for the soil (3 mg/kg for Cu and 23 mg/kg for Zn) at a sorbent dose of 2.5%. This indicates the ability of applied sorbents to firmly bind metals on their surface and reduce their availability in contaminated soils [11].

The greatest decrease in the availability of metals was observed at a 5-APC dose of metal acetate with 5% GAC and biochar (up to 0.5%–3% of the exchangeable compounds) (Figure 2). Both sorbents showed high efficiency in contaminated soils. The difference among the treatments was generally small (up to 3% between the treatments with the amendments of biochar and GAC).

To assess the availability of metals in the soil, an appropriate indicator is the percentages of exchangeable, complexed, and specifically sorbed forms of metals in the group of loosely bound compounds. In uncontaminated soil, the distribution of Cu forms in the group of loosely bound compounds was in the following order: specifically sorbed > complexed > exchangeable. In the case of Zn contamination, the content of the exchangeable forms was higher than that of complexed compounds (Table 4).

Specifically sorbed compounds played a more significant role in the sorption of Zn. It should be noted that when soil was contaminated, the content of specifically sorbed HM forms decreased (up to 39%–51% of the group of loosely bound compounds), but the content of more mobile forms—exchangeable and complexed—increased. Moreover, the distribution within the group reveals the nature of the metal itself, namely, the prevalence of exchangeable forms of compounds (up to 36%) was characteristic for Zn, whereas complexed forms bound with soil organic matter (up to 33%) were characteristic of Cu (Table 4).

The application of both biochar and GAC on contaminated soil resulted in the recovery of the ratio of metal compounds in the set of loosely bound compounds to the level characteristic of uncontaminated soil. The most effective dose was 5% for both biochar and GAC applications (Table 4).

### 3.2. Cu and Zn Accumulation in Spring Barley

One of the criteria for assessing the level of soil pollution with HMs is determining their influence on plant growth and development. The presence in the soil of a large number of bioavailable forms of HMs risks their entry into plants and, subsequently, through the food chain, both into animals and humans, thereby posing a threat to the life and health of society.

The study of HM content allowed us to establish significant differences in element accumulation (Table 3). In the control plants, the content of Cu and Zn in spring barley grain did not exceed their MPC (10 and 50 mg/kg, respectively) (Table 5) [68].

Concentrations of Cu and Zn in spring barley were observed to increase with the increase in the metal content in the soil. The metal content in the underground organs of plants was higher than in the above-ground parts. At a high dose (10 APC) of Cu and Zn input to the soil, the concentration of metals in the roots of spring barley increased by 46- and 50-fold, respectively, compared to the control (Table 5). It was evident that Cu absorbed by spring barley from polluted soil could translocate to above-ground tissues up to 40% [69]. A higher accumulation of Cu was observed in spring barley roots (127 mg/kg) than the above-ground tissues (30.2 mg/kg) grown in Cu-spiked soil [70]. The study performed in a hydroponic system showed a higher accumulation of Cu^2+^ content in the spring barley roots (1142 mg/kg) than the shoots (842 mg/kg) [71]. In contrast, a high accumulation of Zn (up to 11,283 mg/kg) was accumulated in spring barley shoots grown in pots [72]. The roots serve as the first barrier against pollutant penetration into the plant from soil pollution [3]. Moreover, the increase in the metal concentration in stems was 11 times with Cu input and 17 times with Zn input, and in the grain the increase was four times and 10 times, respectively (Table 5). It was established that spring barley generative organs demonstrated metal contamination (1 MPC) at a dose of 5 APC Cu. The content of Cu and Zn in spring barley grains exceeded 3 MPC for Cu and 2 MPC for Zn at a pollution dose of 10 APC. Increased content of HMs in plants can be reflected in morphological changes and changes in anatomical indices [47,73]. It was established that HM bioaccumulation in spring barley affected the physiological and ultrastructural changes in plants grown on Technosols. However, the application of biochar impaired the toxic effects of HMs and showed insignificant changes in the maximal quantum yield of photosystem II (one of the most commonly used photosynthesis measuring parameters) compared to plants grown on clean soil [74]. For example, soil amended with biochar showed a significantly higher biomass of maize than the control and plants grown on non-amended soils [75]. Biochar amendments could have profound effects on plant nutrient uptake in HM polluted soil; however, the availability of several plant nutrients depends on the soil pH [76].

The obtained data show that the inactivation of metals in the soil upon the application of sorbents caused a decrease in the accumulation of Cu and Zn in spring barley (Table 5). The efficiency of the use of sorbents on polluted soils is due to their reduction in the content of HM in the grain below the MPC level. With a soil contamination of 5 APC Cu, the effective dose was 2.5% biochar and GAC, with pollution of 10 APC the effective dose was 5% biochar and GAC. In the case of Zn, contamination of the generative organs of spring barley was observed only at a dose of 10 APC. Here, a dose of 5% biochar and GAC was effective. The greatest decrease in the content of HM in plants was obtained at doses of 5% of both biochar and GAC for any contamination. There was no significant difference between biochar and GAC. In this case, the amount of Cu in the roots dropped by eight times, in the stems up to five times, and in the grain up to three times, with a soil contamination of 5 APC Cu. The same trends were observed when using sorbents in Zn contamination (10 APC). The metal content decreased in the roots up to five times, in the stems up to six times, and in the grain up to four times.

The accumulation coefficient (AC) made it possible to assess the intensity of HM entry into spring barley from the soil (Table 5). The intensity of Cu and Zn accumulation by spring barley tissues from unpolluted soil (control) was very high (AC > 1), which can be associated with their important role as essential elements in plants and also indicates that the plant is a good metal accumulator (Table 5). The AC of Zn in spring barley decreased sharply when the metal was introduced into the soil. However, as the level of Zn pollution increased, it did not change (AC = 0.7). In the case of Cu, the absorption intensity in the variant with 5 APC HM remained high (AC = 1), and with an increase in the metal concentration in the soil (10 APC HM) decreased (AC = 0.8), which indicates the manifestation of the barrier functions of plants.

When applying sorbents at the 5 APC level of HM soil contamination, an increase in AC was observed, which was due to the immobilization of metals in the soil in the presence of sorbents. In this case, Cu and Zn played the role of essential elements for spring barley. At the 10 APC level of HM soil contamination when applying sorbents, a decrease in AC was observed, which indicates a decrease in the number of HM pollutants entering the plant roots. The lowest AC values were observed in the variants with a 5% dose of biochar and GAC added to contaminated soils. The sorbents/soil amendments could reduce the availability of metal ions through various processes such as adsorption, complexation, precipitation, dissolution and redox reactions, and toxicity [77,78,79]. Thus, biochar was used to improve soil fertility and crop production under normal and stressed conditions.

## 4. Conclusions

The experimental results indicated that the addition of carbon sorbents is an effective method for the remediation of Zn- and Cu-contaminated Haplic Chernozem soil. The share of loosely bound compounds of HMs decreased with the introduction of carbon sorbents.

The accumulation coefficient (AC) made it possible to assess the intensity of HM entry into spring barley from the soil (Table 5). The intensity of Cu and Zn accumulation by spring barley tissues from unpolluted soil was very high (AC > 1) and decreased sharply when the metal was introduced into the soil. The content of Cu and Zn in spring barley grain exceeded MPC for Cu and Zn under the soil pollution conditions.

The application of carbon sorbents into the contaminated soil resulted in the immobilization of loosely bound metal compounds (exchangeable, complexed, and specifically sorbed forms) due to their highly porous structure. With an increase in the dose of sorbents, the inactivating effect was increased. Despite the great complexity and cost in the production of GAC, a comparable level of efficiency was achieved with the use of biochar. The amendments containing biochar or GAC can be useful in decreasing Cu and Zn uptake by spring barley. For all amendments, the concentration of Cu and Zn in all parts of plants was decreased compared with unamended soil. The effect of the use of sorbents on polluted soils is to reduce the content of Cu and Zn in the barley grain below the MPC level. There was no significant difference between biochar and GAC. The most effective doses were 2.5% and 5% of carbon sorbents in soil contaminations of 5 APC and 10 APC of Cu or Zn for both biochar and GAC applications.

## Figures and Tables

**Table 1:** The scheme of the experimental treatments.

Treatment	Pollution
Control	Soil without pollution
Cu	660 mg/kg for Cu (5 APC *)
Cu + 1% sorbent **	660 mg/kg for Cu (5 APC)
Cu + 2.5% sorbent	660 mg/kg for Cu (5 APC)
Cu + 5% sorbent	660 mg/kg for Cu (5 APC)
Zn	1100 mg/kg Zn (5 APC)
Zn + 1% sorbent	1100 mg/kg Zn (5 APC)
Zn + 2.5% sorbent	1100 mg/kg Zn (5 APC)
Zn + 5% sorbent	1100 mg/kg Zn (5 APC)
Cu	1320 mg/kg Cu (10 APC)
Soil + 1% sorbent	1320 mg/kg Cu (10 APC)
Soil + 2.5% sorbent	1320 mg/kg Cu (10 APC)
Soil + 5% sorbent	1320 mg/kg Cu (10 APC)
Zn	2200 mg/kg Zn (10 APC)
Zn + 1% sorbent	2200 mg/kg Zn (10 APC)
Zn + 2.5% sorbent	2200 mg/kg Zn (10 APC)
Zn + 5% sorbent	2200 mg/kg Zn (10 APC)

* APC—approximate permissible concentrations for the total heavy metal content. ** Biochar/GAC—granular activated carbon.

**Table 2:** Elemental composition, specific surface area, and porosity of the used carbonaceous sorbents.

Sorbents	Content of Elements and Ash, %					Atomic Ratio of Elements			Water Content, %	pH	Specific Surface Area, m^2^ g^−1^	Volume of Pores, cm^3^ g^−1^			
	C	N	H	O	Ash	H/C	O/C	(N + O)/C				Total	Micro	Meso	Macro
GAC*	72.5	2.0	3.2	4.3	18.0	0.53	0.05	0.07	3.2	9.3	766	0.82	0.49	0.17	0.16
Biochar	73.7	2.3	2.7	6.1	15.2	0.44	0.06	0.09	3.6	9.1	624	0.85	0.60	0.04	0.21

* GAC–granular activated carbon.

**Table 3:** Total content and concentration of exchangeable, complexed and specifically sorbed Cu and Zn compounds in the Haplic Chernozem soil when applying different doses of metals, mg/kg.

Treatment	Loosely Bound Compounds			Total Content
	Exchangeable	Complexed	Specifically Sorbed	
Cu				
Control	0.6 ± 0.1 *	1.4 ± 0.1	2.2 ± 0.2	45.3
Cu 5 APC **	59.7 ± 5.4	70.2 ± 6.3	106.8 ± 9.6	692.0
Cu 5 APC +1% GAC ***	46.2 ± 4.2	48.1 ± 4.3	91.7 ± 8.3	680.0
Cu 5 APC +1% biochar	36.5 ± 3.3	44.2 ± 4.0	72.1 ± 6.5	691.0
Cu 5 APC +2.5% GAC	5.9 ± 0.5	17.2 ± 1.5	37.1 ± 3.3	684.0
Cu 5 APC +2.5% biochar	5.6 ± 0.5	14.5 ± 1.3	25.9 ± 2.3	696.0
Cu 5 APC +5% GAC	3.0 ± 0.3	10.3 ± 0.9	19.3 ± 1.7	691.0
Cu 5 APC +5% biochar	2.1 ± 0.2	9.3 ± 0.8	17.4 ± 1.6	678.0
Cu 10 APC	197.5 ± 17.8	237.8 ± 21.4	278.8 ± 25.1	1371.0
Cu 10 APC +1% GAC	158.1 ± 14.2	161.7 ± 14.6	250.0 ± 22.5	1370.0
Cu 10 APC +1% biochar	129.2 ± 11.6	142.7 ± 12.8	240.1 ± 21.6	1380.0
Cu 10 APC +2.5% GAC	82.0 ± 7.4	131.0 ± 11.8	245.0 ± 22.0	1373.0
Cu 10 APC +2.5% biochar	67.8 ± 6.1	101.4 ± 9.1	216.1 ± 19.4	1369.0
Cu 10 APC +5% GAC	41.0 ± 3.7	72.5 ± 6.5	173.8 ± 15.6	1367.0
Cu 10 APC +5% biochar	35.1 ± 3.2	57.8 ± 5.2	142.9 ± 12.9	1375.0
	MPC 3.0 **** [62]			APC 132.0 ** [41]
**Zn**				
Control	2.3 ± 0.2	1.1 ± 0.1	9.6 ± 0.9	84.3
Zn 5 APC	162.1 ± 14.6	67.8 ± 6.1	243.5 ± 21.9	1182.0
Zn 5 APC +1% GAC	134.1 ± 12.1	55.0 ± 4.9	191.6 ± 17.2	1180.0
Zn 5 APC +1% biochar	119.2 ± 10.7	39.5 ± 3.6	182.5 ± 16.4	1183.0
Zn 5 APC +2.5% GAC	63.5 ± 5.7	25.8 ± 2.3	136.3 ± 12.3	1179.0
Zn 5 APC +2.5% biochar	67.9 ± 6.1	20.9 ± 1.9	123.1 ± 11.1	1178.0
Zn 5 APC +5% GAC	9.7 ± 0.9	6.9 ± 0.6	35.1 ± 3.2	1175.0
Zn 5 APC +5% biochar	8.3 ± 0.7	4.6 ± 0.4	31.1 ± 2.8	1185.0
Zn 10 APC	475.6 ± 42.8	220.1 ± 19.8	607.7 ± 54.7	2289.0
Zn 10 APC +1% GAC	368.2 ± 33.1	181.8 ± 16.4	534.7 ± 48.1	2288.0
Zn 10 APC +1% biochar	335.4 ± 30.2	153.5 ± 13.8	494.4 ± 44.5	2280.0
Zn 10 APC +2.5% GAC	220.3 ± 19.8	100.1 ± 9.0	414.3 ± 37.3	2281.0
Zn 10 APC +2.5% biochar	208.3 ± 18.7	94.9 ± 8.5	382.6 ± 34.4	2277.0
Zn 10 APC +5% GAC	100.8 ± 9.1	64.9 ± 5.8	316.7 ± 28.5	2284.0
Zn 10 APC +5% biochar	89.5 ± 8.1	58.2 ± 5.2	307.5 ± 27.7	2284.0
	MPC 23.0 **** [62]			APC 220.0 ** [41]

* Values represented are averages ± standard deviation; the number of replicates corresponded to three (*n* = 3). ** APC—approximate permissible concentrations for the total heavy metal (HM) content. *** GAC–granular activated carbon. **** MPC—maximum permissible concentrations for exchangeable HM compounds.

**Table 4:** The percentage of Cu and Zn in the group of loosely bound compounds.

Treatment	% from Loosely Bound Compounds		
	Exchangeable	Complexed	Specifically Sorbed
Cu			
Control	14	33	53
Cu 5 APC *	25	30	45
Cu 5 APC + 1% GAC **	25	26	49
Cu 5 APC + 1% biochar	24	29	47
Cu 5 APC + 2.5% GAC	10	29	61
Cu 5 APC + 2.5% biochar	12	31	57
Cu 5 APC + 5% GAC	9	32	59
Cu 5 APC + 5% biochar	7	32	61
Cu 10 APC	28	33	39
Cu 10 APC + 1% GAC	28	28	44
Cu 10 APC + 1% biochar	25	28	47
Cu 10 APC + 2.5% GAC	18	29	53
Cu 10 APC + 2.5% biochar	18	26	56
Cu 10 APC + 5% GAC	14	25	61
Cu 10 APC + 5% biochar	15	24	61
**Zn**			
Control	18	8	74
Zn 5 APC	34	14	52
Zn 5 APC + 1% GAC	35	14	51
Zn 5 APC + 1% biochar	35	12	53
Zn 5 APC + 2.5% GAC	28	11	61
Zn 5 APC + 2.5% biochar	32	10	58
Zn 5 APC + 5% GAC	19	13	68
Zn 5 APC + 5% biochar	19	10	71
Zn 10 APC	36	17	47
Zn 10 APC + 1% GAC	34	17	49
Zn 10 APC + 1% biochar	34	16	50
Zn 10 APC + 2.5% GAC	30	14	56
Zn 10 APC + 2.5% biochar	30	14	56
Zn 10 APC + 5% GAC	21	13	66
Zn 10 APC + 5% biochar	20	12	68

* APC—approximate permissible concentrations for the total heavy metal content. ** GAC–granular activated carbon.

**Table 5:** The content of Cu and Zn in spring barley (*Hordeum sativum* distichum), mg/kg.

Treatment	Cu Content in			AC * Cu	Zn Content in			AC Zn
	Root	Stem	Grain		Root	Stem	Grain	
Control	12.7 ± 1.1 **	10.4 ± 0.9	7.0 ± 0.6	3.0	19.20 ± 1.7	17.4 ± 1.6	10.4 ± 0.9	1.5
HM *** 5 APC ****	243.6 ± 21.9	67.1 ± 6.0	12.3 ± 1.1	1.0	348.5 ± 31.4	85.8 ± 7.7	33.2 ± 3.0	0.7
HM 5 APC + 1% GAC *****	177.8 ± 16.0	52.3 ± 4.7	10.0 ± 0.9	1.0	265.8 ± 23.9	64.5 ± 5.8	31.1 ± 2.8	0.7
HM 5 APC + 1% biochar	167.2 ± 15.0	56.2 ± 5.1	9.5 ± 0.9	1.1	263.6 ± 23.7	61.3 ± 5.5	30.8 ± 2.8	0.8
HM 5 APC + 2.5% GAC	108.8 ± 9.8	27.7 ± 2.5	8.4 ± 0.8	1.8	176.2 ± 15.9	42.8 ± 3.9	25.3 ± 2.3	0.8
HM 5 APC + 2.5% biochar	100.3 ± 9.0	25.3 ± 2.3	8.2 ± 0.7	2.2	158.4 ± 14.3	35.2 ± 3.2	23.6 ± 2.1	0.7
HM 5 APC + 5% GAC	60.5 ± 5.4	14.5 ± 1.3	7.3 ± 0.7	1.9	75.3 ± 6.8	29.6 ± 2.7	16.5 ± 1.5	1.5
HM 5 APC + 5% biochar	59.9 ± 5.4	14.2 ± 1.3	6.9 ± 0.6	2.1	63.4 ± 5.7	28.8 ± 2.6	16.9 ± 1.5	1.4
HM 10 APC	578.2 ± 52.0	116.3 ± 10.5	27.2 ± 2.4	0.8	956.8 ± 86.1	286.5 ± 25.8	98.5 ± 8.9	0.7
HM 10 APC + 1% GAC	365.3 ± 32.9	68.0 ± 6.1	24.8 ± 2.2	0.6	627.0 ± 56.4	241.5 ± 21.7	86.9 ± 7.8	0.6
HM 10 APC + 1% biochar	349.9 ± 31.5	65.5 ± 5.9	23.9 ± 2.2	0.7	608.3 ± 54.7	239.8 ± 21.6	81.4 ± 7.3	0.6
HM 10 APC + 2.5% GAC	177.4 ± 16.0	35.5 ± 3.2	11.5 ± 1.0	0.4	325.0 ± 29.3	146.8 ± 13.2	55.3 ± 5.0	0.4
HM 10 APC + 2.5% biochar	166.9 ± 15.0	31.6 ± 2.8	11.4 ± 1.0	0.4	307.0 ± 27.6	102.5 ± 9.2	49.2 ± 4.4	0.4
HM 10 APC + 5% GAC	73.0 ± 6.6	22.4 ± 2.0	8.6 ± 0.8	0.3	204.0 ± 18.4	47.4 ± 4.3	23.1 ± 2.1	0.4
HM 10 APC + 5% biochar	68.6 ± 6.2	20.7 ± 1.9	7.1 ± 0.6	0.3	193.0 ± 17.4	43.6 ± 3.9	22.5 ± 2.0	0.4

* AC—accumulation coefficient. ** Values represented are averages ± standard deviation; the number of replicates corresponded to three (*n* = 3). *** HM—heavy metal. **** APC—approximate permissible concentrations for the total HM content. ***** GAC–granular activated carbon.

## Data Availability

Data is contained within the article.
